# Supplementary material for: MHC class I allele diversity in cynomolgus macaques of Vietnamese origin
Source: PeerJ. 2019 Nov 4;7:e7941. doi: 10.7717/peerj.7941 (PMC6836755; doi:10.7717/peerj.7941)
Supplement: Figure S1 [file peerj-07-7941-s001.pdf]

Figure S1

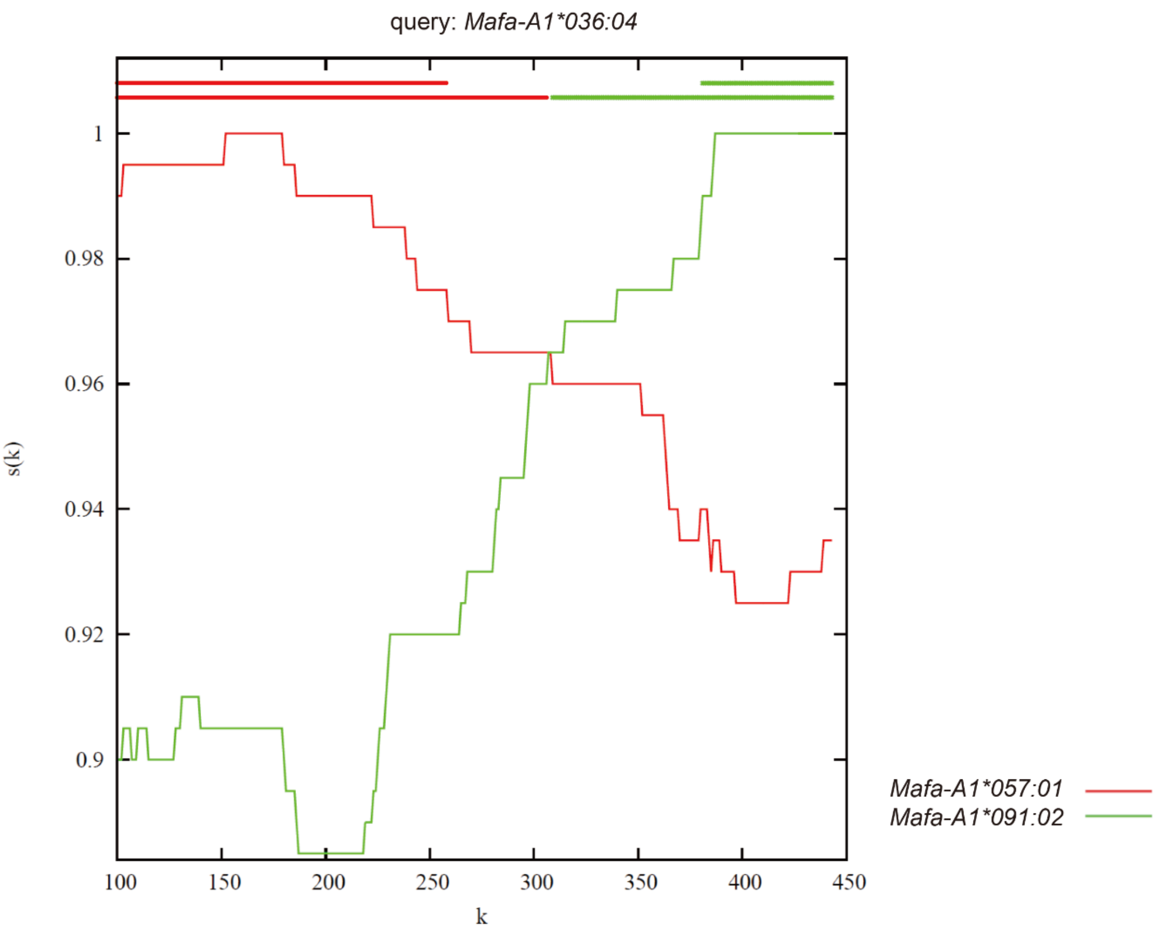

Figure S1. Schematic presentation of a putative crossover event between *Mafa-A1*091:02* and *Mafa-A1*057:01*, by which *Mafa-A1*036:04* was generated.
