# Supplementary material for: MHC class I allele diversity in cynomolgus macaques of Vietnamese origin
Source: PeerJ. 2019 Nov 4;7:e7941. doi: 10.7717/peerj.7941 (PMC6836755; doi:10.7717/peerj.7941)
Supplement: Table S1 [file peerj-07-7941-s002.docx]

**Table S1** Recombination test using the RDP program

|  | **recombinant sequence** | **Breakpoint Positions** | **Major parent** | **Minor parent** | **Methods** |
| --- | --- | --- | --- | --- | --- |
|  |  | **Begin End** |  |  |  |
| 1 | *Mafa-A1*036:04* | 305 521* | *Mafa-A1*057:01* | *Mafa-A1*091:02* | B M C S T |
| 2 | *Mafa-A1*043:01* | 290 517* | *Mafa-A4*14:09* | *Mafa-A1*032:03:02* | B M C S |
| 3 | *Mafa-A1*079:02* | 16* 210 | *Mafa-A1*066:06* | *Mafa-A3*13:02* | M C S T |
| 4 | *Mafa-B*001:01:01* | 16* 184 | *Mafa-B*015:03* | *Mafa-B*021:02* | B M S T |

* means the actual breakpoint position is undetermined.

G: GENECONV, B: BootScan, M: MaxChi, C: Chimaera, S: SiScan, T: 3Seq.
