# Supplementary material for: MHC class I allele diversity in cynomolgus macaques of Vietnamese origin
Source: PeerJ. 2019 Nov 4;7:e7941. doi: 10.7717/peerj.7941 (PMC6836755; doi:10.7717/peerj.7941)
Supplement: Table S2 [file peerj-07-7941-s003.docx]

| **Table S2** Nucleotide diversity of *Mafa-A1* exons 2 and 3 sequences | | | | |
| --- | --- | --- | --- | --- |
| Category | Positions | Nucleotides at each position | Frequency for the most common nucleotide | Frequency for the second-most common nucleotide |
| rare variable site | 2 | C G | 95.52% | 4.48% |
| informative sites | 5 | C G T | 50.75% | 40.30% |
| informative sites | 12 | A T | 85.07% | 14.93% |
| rare variable site | 16 | G A | 95.52% | 4.48% |
| informative sites | 17 | G C A | 80.60% | 14.93% |
| informative sites | 24 | T C A | 91.04% | 7.46% |
| rare variable site | 25 | A G | 98.51% | 1.49% |
| rare variable site | 26 | C T | 98.51% | 1.49% |
| informative sites | 30 | T G A | 76.12% | 22.39% |
| informative sites | 31 | C A T | 82.09% | 14.93% |
| informative sites | 33 | A G | 59.70% | 40.30% |
| rare variable site | 34 | T A | 98.51% | 1.49% |
| informative sites | 44 | C T | 88.06% | 11.94% |
| rare variable site | 45 | G C | 98.51% | 1.49% |
| rare variable site | 50 | C T | 98.51% | 1.49% |
| rare variable site | 51 | G T | 97.01% | 2.99% |
| informative sites | 54 | C G | 64.18% | 35.82% |
| informative sites | 66 | A T G | 68.66% | 16.42% |
| informative sites | 68 | C G | 82.09% | 17.91% |
| informative sites | 69 | G T A | 53.73% | 37.31% |
| rare variable site | 86 | C T | 98.51% | 1.49% |
| rare variable site | 99 | G C | 98.51% | 1.49% |
| rare variable site | 102 | C T | 98.51% | 1.49% |
| rare variable site | 103 | G A | 98.51% | 1.49% |
| rare variable site | 104 | G A | 98.51% | 1.49% |
| informative sites | 121 | A C | 50.75% | 49.25% |
| informative sites | 125 | T C | 58.21% | 41.79% |
| informative sites | 127 | C A | 58.21% | 41.79% |
| informative sites | 128 | G A | 85.07% | 14.93% |
| informative sites | 131 | G A | 55.22% | 44.78% |
| informative sites | 132 | A G C | 61.19% | 37.31% |
| informative sites | 133 | T A | 55.22% | 44.78% |
| rare variable site | 138 | C T | 98.51% | 1.49% |
| rare variable site | 139 | C T | 98.51% | 1.49% |
| rare variable site | 155 | G A | 98.51% | 1.49% |
| informative sites | 167 | G T | 64.18% | 35.82% |
| informative sites | 170 | G A | 73.13% | 26.87% |
| informative sites | 182 | C A | 94.03% | 5.97% |
| informative sites | 183 | C T G | 80.60% | 13.43% |
| informative sites | 184 | G A C | 70.15% | 16.42% |
| informative sites | 186 | A G C | 43.28% | 43.28% |
| informative sites | 187 | A G | 94.03% | 5.97% |
| informative sites | 188 | G C | 56.72% | 43.28% |
| rare variable site | 189 | A G | 98.51% | 1.49% |
| rare variable site | 193 | G A | 97.01% | 2.99% |
| rare variable site | 195 | A T | 98.51% | 1.49% |
| informative sites | 196 | T A | 80.60% | 19.40% |
| informative sites | 197 | C A | 92.54% | 7.46% |
| informative sites | 198 | T A G | 47.76% | 46.27% |
| informative sites | 199 | T C A G | 46.27% | 23.88% |
| informative sites | 200 | C G A | 52.24% | 46.27% |
| informative sites | 204 | G A | 74.63% | 25.37% |
| rare variable site | 205 | C A G | 94.03% | 4.48% |
| informative sites | 207 | G A | 74.63% | 25.37% |
| informative sites | 208 | A C | 80.60% | 19.40% |
| informative sites | 209 | G C | 56.72% | 43.28% |
| rare variable site | 210 | A G | 95.52% | 4.48% |
| rare variable site | 214 | A T | 98.51% | 1.49% |
| rare variable site | 216 | A G | 98.51% | 1.49% |
| informative sites | 217 | C A G T | 49.25% | 46.27% |
| informative sites | 218 | C T A G | 65.67% | 28.36% |
| informative sites | 219 | T G C | 67.16% | 31.34% |
| informative sites | 220 | A C T | 67.16% | 31.34% |
| rare variable site | 222 | C A | 97.01% | 2.99% |
| informative sites | 223 | G C | 68.66% | 31.34% |
| informative sites | 226 | A T G C | 55.22% | 40.30% |
| informative sites | 228 | A G | 70.15% | 29.85% |
| informative sites | 229 | G A C | 41.79% | 40.30% |
| rare variable site | 234 | C A G | 95.52% | 2.99% |
| informative sites | 235 | G A | 91.04% | 8.96% |
| informative sites | 238 | A C T | 62.69% | 29.85% |
| informative sites | 240 | C G | 89.55% | 10.45% |
| informative sites | 241 | T C | 89.55% | 10.45% |
| informative sites | 242 | G T A | 76.12% | 22.39% |
| informative sites | 244 | G T | 76.12% | 23.88% |
| informative sites | 246 | G C | 76.12% | 23.88% |
| rare variable site | 264 | G C | 98.51% | 1.49% |
| rare variable site | 268 | C G | 97.01% | 2.99% |
| informative sites | 269 | C T G | 86.57% | 7.46% |
| rare variable site | 278 | C T | 98.51% | 1.49% |
| informative sites | 282 | A T C G | 37.31% | 35.82% |
| informative sites | 283 | T A | 76.12% | 23.88% |
| informative sites | 288 | A G T | 91.04% | 5.97% |
| informative sites | 289 | G A C T | 46.27% | 20.90% |
| rare variable site | 290 | G A | 95.52% | 4.48% |
| informative sites | 294 | T G C | 86.57% | 10.45% |
| informative sites | 295 | A T | 85.07% | 14.93% |
| informative sites | 296 | C T | 83.58% | 16.42% |
| informative sites | 311 | G A | 74.63% | 25.37% |
| informative sites | 314 | C A G | 89.55% | 8.96% |
| rare variable site | 315 | G A | 98.51% | 1.49% |
| rare variable site | 321 | C G | 98.51% | 1.49% |
| rare variable site | 325 | T G | 98.51% | 1.49% |
| rare variable site | 326 | C A T | 97.01% | 1.49% |
| rare variable site | 327 | C G | 97.01% | 2.99% |
| rare variable site | 331 | G A | 98.51% | 1.49% |
| rare variable site | 332 | C G | 98.51% | 1.49% |
| informative sites | 335 | G A | 92.54% | 7.46% |
| rare variable site | 336 | T C | 98.51% | 1.49% |
| rare variable site | 338 | T C | 97.01% | 2.99% |
| informative sites | 339 | G A C T | 73.13% | 13.43% |
| informative sites | 340 | A G T | 85.07% | 13.43% |
| informative sites | 341 | C A G | 53.73% | 44.78% |
| informative sites | 345 | T C G | 88.06% | 8.96% |
| informative sites | 346 | A C T | 34.33% | 32.84% |
| informative sites | 361 | G A | 58.21% | 41.79% |
| rare variable site | 362 | G A T | 97.01% | 1.49% |
| rare variable site | 369 | A T | 95.52% | 4.48% |
| rare variable site | 372 | G T | 98.51% | 1.49% |
| rare variable site | 381 | G C | 98.51% | 1.49% |
| informative sites | 382 | A G | 86.57% | 13.43% |
| informative sites | 383 | G C | 89.55% | 10.45% |
| rare variable site | 390 | C A | 98.51% | 1.49% |
| informative sites | 392 | C T | 83.58% | 16.42% |
| informative sites | 401 | C G A | 56.72% | 40.30% |
| informative sites | 404 | C A G T | 76.12% | 11.94% |
| rare variable site | 410 | C T | 98.51% | 1.49% |
| informative sites | 411 | A T G C | 73.13% | 19.40% |
| informative sites | 412 | T C | 88.06% | 11.94% |
| rare variable site | 415 | C G | 98.51% | 1.49% |
| rare variable site | 422 | G A | 98.51% | 1.49% |
| informative sites | 434 | G C | 76.12% | 23.88% |
| rare variable site | 443 | G A | 98.51% | 1.49% |
| rare variable site | 445 | C T | 98.51% | 1.49% |
| informative sites | 448 | C A | 94.03% | 5.97% |
| informative sites | 449 | G C A | 77.61% | 17.91% |
| rare variable site | 450 | G C | 95.52% | 4.48% |
| informative sites | 451 | G A | 91.04% | 8.96% |
| informative sites | 453 | G T | 82.09% | 17.91% |
| informative sites | 454 | T C A G | 40.30% | 26.87% |
| rare variable site | 456 | G A | 95.52% | 4.48% |
| rare variable site | 458 | G A T | 95.52% | 2.99% |
| informative sites | 462 | C A | 94.03% | 5.97% |
| informative sites | 463 | A G C | 76.12% | 20.90% |
| rare variable site | 464 | G C | 95.52% | 4.48% |
| informative sites | 465 | A T C G | 37.31% | 25.37% |
| informative sites | 466 | T A G | 52.24% | 34.33% |
| informative sites | 467 | C G | 56.72% | 43.28% |
| informative sites | 471 | G A | 68.66% | 31.34% |
| informative sites | 472 | C T | 89.55% | 10.45% |
| informative sites | 477 | C G | 92.54% | 7.46% |
| informative sites | 484 | G C | 92.54% | 7.46% |
| rare variable site | 485 | C G | 98.51% | 1.49% |
| informative sites | 486 | G C A | 64.18% | 26.87% |
| informative sites | 487 | A G T C | 71.64% | 17.91% |
| informative sites | 488 | G A | 91.04% | 8.96% |
| informative sites | 491 | C T | 88.06% | 11.94% |
| informative sites | 492 | C G | 68.66% | 31.34% |
| informative sites | 499 | G C | 83.58% | 16.42% |
| rare variable site | 504 | C G | 98.51% | 1.49% |
| rare variable site | 505 | G C A | 94.03% | 4.48% |
| informative sites | 510 | T C | 86.57% | 13.43% |
| rare variable site | 512 | C T | 95.52% | 4.48% |
| rare variable site | 521 | C G | 95.52% | 4.48% |
| rare variable site | 533 | G A | 98.51% | 1.49% |
| rare variable site | 541 | G A | 98.51% | 1.49% |
| rare variable site | 543 | G A | 95.52% | 4.48% |
