# Supplementary material for: MHC class I allele diversity in cynomolgus macaques of Vietnamese origin
Source: PeerJ. 2019 Nov 4;7:e7941. doi: 10.7717/peerj.7941 (PMC6836755; doi:10.7717/peerj.7941)
Supplement: Table S3 [file peerj-07-7941-s004.docx]

**Table S3** The diversity of *Mafa-A1* amino acid sequences encoding α1 and α2 domains

| Category | Positions | Residues at each position | Frequency for the most common residue | Frequency for the second-most common residue |
| --- | --- | --- | --- | --- |
| informative site | 5 | M L | 85.07% | 14.93% |
| informative site | 6 | R S K | 80.60% | 14.93% |
| informative site | 9 | Y H S | 91.04% | 7.46% |
| informative site | 11 | S A Y F T | 58.21% | 22.39% |
| informative site | 12 | M V E | 59.70% | 38.81% |
| rare variable site | 16 | G R | 98.51% | 1.49% |
| rare variable site | 18 | G W | 97.01% | 2.99% |
| informative site | 19 | Q E | 64.18% | 35.82% |
| informative site | 23 | I M F V | 50.75% | 17.91% |
| informative site | 24 | A S T | 53.73% | 37.31% |
| rare variable site | 34 | V L | 98.51% | 1.49% |
| rare variable site | 35 | R Q W | 97.01% | 1.49% |
| informative site | 41 | E A | 50.75% | 49.25% |
| informative site | 43 | P Q | 58.21% | 41.79% |
| informative site | 45 | M E K L | 53.73% | 37.31% |
| rare variable site | 47 | P L S | 97.01% | 1.49% |
| informative site | 61 | D E | 94.03% | 5.97% |
| informative site | 62 | R S Q E | 70.15% | 13.43% |
| informative site | 63 | E N Q S | 43.28% | 37.31% |
| rare variable site | 64 | T A | 98.51% | 1.49% |
| rare variable site | 65 | R Q | 97.01% | 2.99% |
| informative site | 66 | I N K Y | 80.60% | 10.45% |
| informative site | 67 | M S Y C A | 46.27% | 17.91% |
| informative site | 69 | A T D S | 70.15% | 23.88% |
| informative site | 70 | E N D A T | 41.79% | 20.90% |
| rare variable site | 71 | T A | 95.52% | 4.48% |
| rare variable site | 72 | Q L | 98.51% | 1.49% |
| informative site | 73 | T N R D K M | 49.25% | 43.28% |
| informative site | 74 | Y A L | 67.16% | 31.34% |
| informative site | 75 | R P | 68.66% | 31.34% |
| informative site | 76 | E V G A | 55.22% | 40.30% |
| informative site | 77 | N S A G D | 38.81% | 31.34% |
| informative site | 79 | R Q G | 89.55% | 8.96% |
| informative site | 80 | N T I | 62.69% | 29.85% |
| informative site | 81 | L A | 89.55% | 10.45% |
| informative site | 82 | R L | 76.12% | 23.88% |
| informative site | 83 | G R | 76.12% | 23.88% |
| rare variable site | 89 | E Q | 98.51% | 1.49% |
| rare variable site | 90 | A G | 97.01% | 2.99% |
| informative site | 95 | I Y L F V | 37.31% | 23.88% |
| informative site | 97 | R K T V M W I | 43.28% | 20.90% |
| informative site | 99 | Y V L F | 85.07% | 10.45% |
| rare variable site | 106 | D N | 98.51% | 1.49% |
| rare variable site | 108 | R G | 98.51% | 1.49% |
| rare variable site | 109 | L R | 98.51% | 1.49% |
| rare variable site | 110 | L V | 97.01% | 2.99% |
| rare variable site | 111 | R H | 98.51% | 1.49% |
| rare variable site | 113 | Y H | 98.51% | 1.49% |
| informative site | 114 | E D S H Y R N V | 43.28% | 28.36% |
| informative site | 116 | F S Y H D | 32.84% | 32.84% |
| informative site | 121 | R K S | 56.72% | 41.79% |
| rare variable site | 124 | I F | 95.52% | 4.48% |
| rare variable site | 125 | A S | 98.51% | 1.49% |
| informative site | 128 | E G D Q | 74.63% | 13.43% |
| rare variable site | 131 | R S | 98.51% | 1.49% |
| informative site | 138 | M L T V | 61.19% | 20.90% |
| rare variable site | 139 | A G | 98.51% | 1.49% |
| rare variable site | 149 | A V | 98.51% | 1.49% |
| informative site | 150 | A E | 94.03% | 5.97% |
| informative site | 151 | G D R | 86.57% | 8.96% |
| informative site | 152 | V A E W L | 38.81% | 26.87% |
| rare variable site | 153 | A T | 95.52% | 4.48% |
| informative site | 155 | Q R S P | 76.12% | 16.42% |
| informative site | 156 | M D F H I W Q R | 25.37% | 16.42% |
| informative site | 158 | A T V | 58.21% | 31.34% |
| informative site | 160 | L V | 92.54% | 7.46% |
| informative site | 162 | G A | 92.54% | 7.46% |
| informative site | 163 | E R L K Q G T | 61.19% | 14.93% |
| informative site | 165 | L V | 68.66% | 31.34% |
| informative site | 167 | W S | 83.58% | 16.42% |
| rare variable site | 169 | R P G H | 92.54% | 4.48% |
| informative site | 171 | Y H | 86.57% | 13.43% |
| rare variable site | 174 | N K | 95.52% | 4.48% |
| rare variable site | 181 | R H | 98.51% | 1.49% |
| rare variable site | 182 | A T | 95.52% | 4.48% |
